# Factors associated with empathy among community pharmacists in Lebanon

**DOI:** 10.1186/s40545-020-00237-z

**Published:** 2020-08-14

**Authors:** Eva Hobeika, Souheil Hallit, Hala Sacre, Sahar Obeid, Aline Hajj, Pascale Salameh

**Affiliations:** 1 Faculty of Arts and Sciences, Holy Spirit University of Kaslik (USEK), Jounieh, Lebanon; 2 INSPECT-LB: Institut National de Santé Publique, Épidémiologie Clinique et Toxicologie, Beirut, Lebanon; 3 Faculty of Medicine and Medical Sciences, Holy Spirit University of Kaslik (USEK), Jounieh, Lebanon; 4 Drug Information Center, Order of Pharmacists of Lebanon, Beirut, Lebanon; 5 Departments of Psychology and Research, Psychiatric Hospital of the Cross, Jal Eddib, Lebanon; 6 Laboratory of Pharmacology, Clinical Pharmacy and Quality Control of Drugs, Faculty of Pharmacy, Pôle Technologie-Santé (PTS), Faculty of Pharmacy, Saint-Joseph University, Beirut, 1107 2180 Lebanon; 7 Faculty of Pharmacy, Saint-Joseph University, Beirut, 1107 2180 Lebanon; 8 Faculty of Pharmacy, Lebanese University, Hadat, Lebanon; 9 Faculty of Medicine, Lebanese University, Hadat, Lebanon

**Keywords:** Empathy, Community pharmacists, Work fatigue, Lebanon

## Abstract

**Introduction:**

Empathy is the cornerstone of the relationship between the healthcare provider and the patient. In Lebanon, no studies have investigated the factors associated with empathy among community pharmacists. Hence, the importance of this research to better understand empathy and help community pharmacists with this vital aspect of their practice.

**Objective:**

This study aimed to evaluate empathy and possible factors associated with it among Lebanese community pharmacists.

**Methods:**

This cross-sectional study was carried out between March and July 2018. It enrolled a proportionate random sample of 435 community pharmacists from all Lebanese districts. The Epi info software calculated the minimum sample size, based on a total number of 3762 community pharmacists, with an expected frequency of 50% of pharmacists with low empathy, and a 95% confidence interval. The minimal sample size required was 350 community pharmacists; our sample size was 435 to account for missing values.

**Results:**

Our results revealed that 228 (53.4%) pharmacists had low empathy. Lower empathy was significantly associated with more physical (Beta = − 0.331) and mental (Beta = − 0.126) work fatigue, higher age (Beta = − 0.125) and a practice experience between 3 years and less than 6 years compared to less than 6 months (Beta = − 2.440).

**Conclusion:**

This study shed the light on some factors associated with empathy among Lebanese community pharmacists. Low empathy levels were significantly associated with factors such as age, practice experience, and mental and physical work fatigue, all of which impact the practice, as the accepted model of pharmacy practice requires that pharmacists establish effective communication and use interpersonal skills. Therefore, developing empathetic communication skills is considered essential. Furthermore, increased mental and physical work fatigue should not hinder community pharmacists’ access to self-care, whether for their mental or physical health.

## Introduction

Empathy is an ambiguous concept [1] that has been portrayed as a notion hard to define and measure [2]. Multiple definitions exist, but the one adapted for the patient care context is: “*predominantly a cognitive attribute that involves an understanding of patients’ concerns, the capacity to communicate this understanding, and an intention to help”* [1, 3]. It is divided into three components: affective or emotional, cognitive, and somatic. In this paper, empathy will refer to emotional empathy.

Previous studies have established that empathy is the cornerstone of the relationship between the healthcare provider and the patient [1, 4]. Indeed, greater empathy was linked to better patient compliance [5, 6], more accurate diagnosis [7] and prognosis [8], and increased patient satisfaction [6]. Among the various categories of healthcare professionals, community pharmacists are considered to be the most accessible ^5^. Previous findings showed that pharmacists who can communicate empathetically build a good rapport with patients, thus improving patient outcomes [9].

Evidence showed a negative association between empathy and burnout, higher empathy being associated with lower levels of burnout [10, 11], noting that burnout includes mental exhaustion, negative attitudes, and physical depletion. Moreover, the increased psychological distress among healthcare providers is related to decreased empathy and, consequently, alters the quality of care provided [12]. Furthermore, higher depressive symptoms have been correlated with lower empathy, suggesting that efforts to reduce depression may improve levels of empathy [13]. Also, good quality of sleep and recreational activities and exercise were also associated with higher empathy [14].

On the other hand, the relationship between empathy and gender showed that women had higher empathy levels compared to men, to a nearly significant degree [15, 16]. Although empathy is essential for patient care, it declines as medical students progress through training [17]. However, evaluations of the relationship between age and empathy among healthcare professionals, reveal that older practitioners have a higher level of empathy, mainly attributed to the maturity acquired over the years [18].

In 1994, Mark H. Davis introduced a social psychological approach to empathy. It suggested that the individual’s empathic abilities can plausibly influence their management of conflict or other relationship-related behaviors. To inclusively define empathy, Davis proposed an organizational model that would take into account the different constructs falling under the broad heading of “empathy” [19]. This organizational model breaks down a typical empathy encounter into an exposure of the observer to the target, followed by a cognitive, affective, and/or behavioral response taking place from the observer’s side [19]. Four related constructs make up the skeletal structure of this model. Antecedents, processes, intrapersonal, and interpersonal outcomes are delineated by associations between them; especially with constructs adjacent to each other. Davis’ model (1994) has been used extensively as the underlying theoretical framework [20], in healthcare-related literature, aiming at understanding the organizational approach to empathy between healthcare providers and patients. It opened the door towards a better understanding of empathetic interactions in the workplace. Moreover, Gerace et al. integrated themes into Davis’ linear model of empathy, delineating nurses’ efforts for empathic communication with patients [20]. This study has also added a double-headed arrow to indicate perspective-taking as a means to regulate nurses’ emotions towards patients (not present in the original model) [20]. Other scales have been used extensively among health professionals, such as the Jefferson Scale of Empathy. However, it does not offer theoretical tools necessary to establish a textbook basis for the works of empathy, as compared to Davis’ model that provides an organizational aspect to the empathic interactions between healthcare providers and patients.

Lebanon is a country with a political instability, especially after the displacement of over a million Syrian refugees since 2012, which had a negative impact on the country economically and socially [21, 22] and created higher xenophobic attitudes among Lebanese in general [23]. Furthermore, community pharmacists are not satisfied financially especially after the drop in the medications prices following the decisions taken by the Ministry of Public Health [23], which added more stressors to their daily life and a lower quality of life [24].

**Fig. 1:**
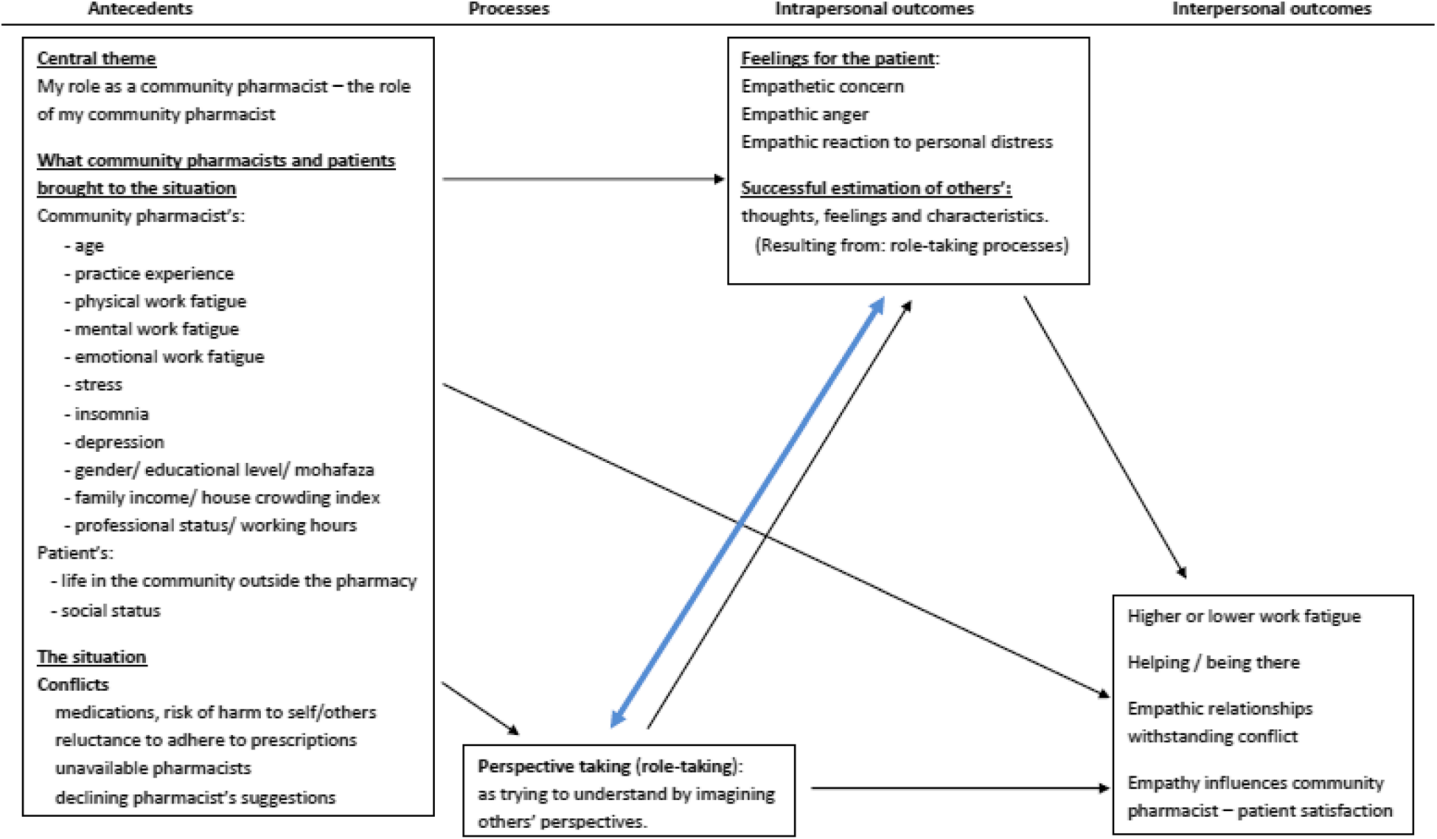
The Organization Model of Empathy in Community Pharmacists. Framework built using Davis’s (1994) linear antecedents, processes, intrapersonal and interpersonal outcomes framework [19]

Based on Davis and Gerace models, Fig. 1 presents an adapted version of the organizational model shedding the light on empathy among community pharmacists. In this figure, the antecedents construct enumerates several factors that community pharmacists have brought to the situation. These factors have the potential to influence both processes and outcomes towards empathy [19] and will be assessed throughout our study.

To the best of our knowledge, no studies have investigated the factors associated with empathy among community pharmacists. Hence, the importance of this research to better understand empathy and help community pharmacists with this vital aspect of their practice.

Therefore, this study aimed to evaluate empathy and possible factors associated with it among Lebanese community pharmacists.

## Methods

### General study design

This cross-sectional study was carried out between March and July 2018. It enrolled a proportionate random sample of 435 community pharmacists from all five Lebanese Mohafazat. Geographically, Lebanon is divided into five major districts, termed Mohafazat, Beirut, North, Mount Lebanon, Beqaa, and South. This sample is based on an exhaustive list provided by the Lebanese Order of Pharmacists (OPL, the official association of pharmacists in Lebanon). The methodology used is described elsewhere [24, 25].

### Sample size calculation

According to the OPL list, a total of 3762 community pharmacists (employers and employees) practice in 3157 community pharmacies distributed across all regions.

The Epi info software calculated the minimum sample size, based on a total number of 3762 community pharmacists, with an expected frequency of 50% of pharmacists with low empathy (in the absence of since similar studies in the country), and a 95% confidence interval. The minimal sample size required was 350 community pharmacists.

Out of the 500 randomly distributed questionnaires (in 500 pharmacies), 435 (87%) were completed and collected back; the remaining 65 (13%) corresponded to pharmacists who refused to participate in this study. Thus, the total sample included 435 participants.

### Questionnaire and variables

The questionnaire was self-administered, closed-ended, and available in either French or English, the teaching languages in Lebanese schools of pharmacy. Well-trained field workers distributed it to the pharmacists after explaining the study objectives and obtaining written informed consent. To ensure optimal objectivity, pharmacists filled out the questionnaire without getting any guidance on any of the questions. The average completion time was between 15 and 20 min. Field workers collected back the questionnaires and sent them in closed boxes for data entry. This process allowed to preserve pharmacists’ anonymity.

The questionnaire consisted of two sections. The first section included socio-demographic and practice characteristics, i.e., age, gender, demographic area, level of education, years of practice, location of the pharmacy, the approximate number of patients per day, job position, working hours per week. A house crowding index was also assessed. It was calculated by dividing the total number of individuals living in the house by the total number of rooms, excluding bathrooms and kitchen.

The second section included the following scales:

#### Toronto empathy questionnaire

It consists of 16 items [26]. A five-point scale, ranging between “never” and “often”, is used to rate each item. Positively worded items 1, 3, 5, 6, 8, 9, 13, and 16 were scored as: 0 (Never); 1 (Rarely); 2 (Sometimes); 3 (Often); 4 (Always), whereas negatively worded items 2, 4, 7, 10, 11, 12, 14 and 15 were reversed. All the scores were summed to derive the total empathy score. Higher scores designated higher empathy. The Cronbach’s alpha in this study was 0.729.

#### The three-dimensional work fatigue inventory (3D-WFI)

This inventory consists of 18 questions divided into three 6-question packs. Each pack measured one dimension of work fatigue: physical (e.g., feeling physical exhaustion at the end of the workday), mental (e.g., facing difficulty to think and concentrate at the end of the workday), and emotional (e.g., facing difficulty to show and deal with emotions at the end of the workday) [27]. The score ranged from 0 (never) to 4 (every day). Higher scores indicated higher fatigue in all three dimensions. The Cronbach’s alpha values were 0.880 (physical work fatigue), 0.710 (mental work fatigue), and 0.848 (emotional work fatigue).

#### Hamilton depression rating scale (HDRS)

This 17-item scale is validated in Lebanon [28]. It measures the severity of depressive symptoms (e.g., feelings of guilt, depressed mood, suicide, etc.) [29]. The total score is computed by summing the answers to the 17 questions, with higher scores indicating higher levels of depression. The Cronbach’s alpha value was 0.870 for this study.

#### Beirut distress scale (BDS-22 scale)

This 22-item scale, validated in Lebanon, is used to screen for stress [30]. It assesses six factors in adults, over the past week: demotivation, depressive symptoms, psychosomatic symptoms, mood deterioration, intellectual inhibition, and anxiety. The total score is calculated on a 4-point Likert scale from 0 (not at all) to 3 (all of the time), with higher scores indicating higher levels of stress. In this study, the Cronbach’s alpha was of 0.935.

#### Lebanese insomnia scale (LIS-18)

This scale, validated in Lebanon, is used to screen for insomnia [31]. It consists of 18 items scored on a 5-point Likert scale from 1 (never) to 5 (always). Items 4, 18, and 22 are reversed. Higher scores indicate higher insomnia. In this study, the Cronbach’s alpha was 0.811.

### Forward and back translation procedure

The translation from English into French was carried out by a translator and validated by a healthcare professionals’ expert committee, and a language professional. A backward translation was then performed by a native English-speaking translator, fluent in French and unfamiliar with the notions of the scales. The expert committee compared the back-translated English version to the original one and resolved discrepancies and inconsistencies by consensus. Both versions were piloted on a sample of 20 pharmacists, before launching data collection. The results of the pilot sample were excluded from the final datasheet.

### Statistical analysis

A study-independent person, not involved in the data collection process, performed the data entry. The statistical analysis was done on SPSS version 23. Student t-test was applied to check for associations between the empathy score and dichotomous variables (i.e., gender). The ANOVA test was used to compare means of 3 or more groups (i.e., educational level). Finally, a stepwise linear regression was performed, taking the self-reported empathy score as the dependent variable, and all the variables that showed a significant association in the bivariate analysis as independent variables. A *p*-value was significant when *p* < 0.05.

## Results

### Sociodemographic characteristics of the participants and other parameters

Out of 500 distributed questionnaires, 435 (87%) were completed and collected back. The mean age of the participants was 38.97 ± 11.13 years, and 52% were males (Table 1).

**Table 1:** Sociodemographic and socioeconomic characteristics of the participants (*n* = 435)

Factor	N (%)
**Gender**	
Male	223 (52.0%)
Female	206 (48.0%)
**District**	
Beirut	77 (18.0%)
Mount Lebanon	150 (35.1%)
North	66 (15.5%)
South	48 (11.2%)
Bekaa	48 (11.2%)
**Educational level**	
Bachelor of science	250 (58.4%)
Pharm.D	106 (24.8%)
Masters	60 (14.0%)
PhD	12 (2.8%)
**Professional status**	
Employer	299 (68.7%)
Employee	128 (30.0%)
**Experience**	
Less than 6 months	24 (5.6%)
6 months to 1 year	20 (4.6%)
1 year to less than 3 years	36 (8.4%)
3 years to less than 6 years	64 (14.8%)
6 years to less than 12 years	118 (27.4%)
More than 12 years	169 (39.2%)
**Approximate number of patients seen per day in the pharmacy**	
< 10	3 (0.7%)
10–50	131 (30.8%)
50–100	188 (44.2%)
> 100	103 (24.2%)
**Working hours per week**	
1–16 h per week	27 (6.3%)
17–31 h per week	48 (11.2%)
32–40 h per week	96 (22.3%)
More than 40 h per week	259 (60.2%)
**Social status of the patients**	
Poor	26 (6.1%)
Middle	193 (45.6%)
High	16 (3.8%)
Do not know	185 (43.7%)
**Family income per month**	
< 1000 USD	35 (8.9%)
1000–2000 USD	90 (20.7%)
2000–3000 USD	129 (32.7%)
> 3000 USD	140 (35.5%)
**Mean ± SD**	
Age (in years)	38.97 ± 11.13
House crowding index	0.89 ± 0.44

Since the Toronto empathy scale does not have a cut-off point, the median (= 60) was used as the cut-off point. The mean empathy score was 59.02 ± 7.32, with 228 (53.4%) of the pharmacists having low empathy. Table 2 summarizes the scores of the intended measured parameters.

**Table 2:** Description of the intended measured parameters in our sample

Variables	Mean ± SD
Empathy score	59.02 ± 7.32
Stress score	42.37 ± 13.49
Insomnia score	37.53 ± 8.44
Depression score	6.90 ± 7.01
Emotional work fatigue score	17.38 ± 10.42
Mental work fatigue score	8.36 ± 6.50
Physical work fatigue score	7.63 ± 8.29

### Bivariate analysis

The results of the bivariate analysis related to the factors associated with the empathy score are summarized in Tables 3 and 4. The mean empathy score was slightly higher in females compared to males (59.95 vs. 58.17; *p* = 0.013). The mean empathy score was significantly higher in those having a Pharm.D degree (59.94) versus all other education levels, in those living in South Lebanon (61.41) compared to all other districts, and in those having a practice experience of fewer than 6 months. Lower empathy was significantly associated with older age (r = − 0.135), higher mental (r = − 0.401) and physical (r = − 0.399) work fatigue, higher insomnia (r = − 0.098), and higher stress (r = − 0.233).

**Table 3:** Bivariate analysis of factors associated with the empathy score

Variable	Mean Empathy Score
**Gender**	
Male	58.17 ± 6.93
Female	59.95 ± 7.61
p-value	**0.013**
**Education level**	
Bachelor of Pharmacy	59.36 ± 6.83
Pharm.D.	59.94 ± 7.65
Master’s degree	56.96 ± 7.78
PhD	56.50 ± 7.30
p-value	**0.038**
**District**	
Beirut	56.85 ± 7.58
Mount Lebanon	59.87 ± 6.89
North	58.28 ± 7.93
South	61.41 ± 5.64
Bekaa	58.72 ± 7.45
p-value	**0.005**
**Years of practice**	
Less than 6 months	63.16 ± 4.87
6 months to less than 1 year	62.15 ± 8.73
One year to less than 3 years	59.25 ± 8.84
3 years to less than 6 years	57.85 ± 7.32
6 years to less than 12 years	59.46 ± 7.69
12 years or more	58.18 ± 6.49
P-value	**0.008**

Post hoc analysis: Districts (Beirut vs Mount Lebanon *p* = 0.033; Beirut vs Bekaa *p* = 0.007); Years of practice (less than 6 months vs 3 years to less than 6 years *p* = 0.034; less than 6 months vs 12 years or more *p* = 0.025)

**Table 4:** Bivariate analysis of continuous variables associated with the empathy score

Age	
r	−0.135
p-value	**0.007**
**Emotional work fatigue**	
r	−0.031
p-value	0.527
**Mental work fatigue**	
r	−0.401
p-value	**< 0.001**
**Physical work fatigue**	
r	−0.399
p-value	**< 0.001**
**Stress score**	
r	−0.233
p-value	**< 0.001**
**Insomnia score**	
r	−0.098
p-value	**0.043**
**Depression score**	
r	−0.077
p-value	0.114

### Multivariable analysis

The results of the stepwise linear regression, taking the empathy score as the dependent variable, revealed that lower empathy was significantly associated with more physical work fatigue (Beta = − 0.331), more mental work fatigue (Beta = − 0.126), a practice experience between 3 years and fewer than 6 years compared to fewer than 6 months (Beta = − 2.440), and older age (Beta = − 0.125) (Table 5).

**Table 5:** Multivariable analysis: Linear regression taking the empathy score as the dependent variable

Variable	Unstandardized Beta	Standardized Beta	***p***	95% Confidence Interval	
Physical work fatigue	−0.331	−0.375	< 0.001	− 0.410	− 0.252
Age	− 0.125	− 0.187	< 0.001	− 0.185	−0.065
Mental work fatigue	−0.126	−0.152	0.015	−0.227	0.025
Years of practice 3 years to less than 6 years compared to less than 6 months compared to less than 6 months^a^	−2.440	−0.122	0.009	−4.275	−0.605

^a^Reference group

## Discussion

A better understanding of empathy leads to a clearer comprehension of the patient-pharmacist interaction and, therefore, to a constructive relationship. The results of the multivariable analysis demonstrated that lower empathy was significantly associated with older age, more physical and mental work fatigue, and a practice experience between 3 years to fewer than 6 years compared to fewer than 6 months.

Our results showed that 53.4% of the Lebanese community pharmacists have low empathy, consistent with those found among American community pharmacists (58% low empathy) [9]. Professional interactions are usually based on objectivity and ethical standards. Hence, the blurring of the line becomes understandable in professions that involve a certain degree of care-giving. Healthcare providers, such as pharmacists, get to a point where they either get too carried out by patients’ concerns, or too little (low empathy). To reach an interactive balance, where empathy is not surpassed by either sympathy or lack of care for patients, pharmacists must learn to manage their empathetic behaviors [19].

In the present study, higher physical and mental work fatigue were associated with lower empathy, in agreement with results from previous research showing that community pharmacists with emotional exhaustion, depersonalization, and average job levels had mental disengagement, and reduced their commitment to helping patients [32–34]. Lebanese community pharmacists, although held in high esteem by the population [35] have a low level of job satisfaction due to financial constraints [36]. This dissatisfaction can explain the fact that pharmacists feel less concerned about communicating with patients and show less empathy towards them. Since the literature establishes a connection between financial constraints and lower empathy, it was used here to hypothesize an explanation for physical and mental work fatigue mediated by financial constraints.

Our results showed that the third dimension of work fatigue, called emotional work fatigue, was not significantly associated with lower empathy. We speculate that emotional work fatigue is affected by several factors, i.e., pharmacist’s state of mind, personality and other environmental cues that are not necessarily related to work performance. Thus, it is not directly affected by one source (the job at hand), like in the case of mental and physical work fatigue.

Previous findings [37] evaluating the correlation between empathy and age among pharmacists from all sectors (not just community pharmacists) have shown that older health professionals had greater empathy and were more assertive. Oppositely, our results revealed that older age was significantly associated with lower empathy; this difference can be explained by the job dissatisfaction experienced by the Lebanese community pharmacists [36]. Through continuous job dissatisfaction, pharmacists become less concerned and caring, expressing lower empathy levels towards their patients. As they get older, their relationship with loyal patients is affected by the lack of time taken to bond and empathize. So, this low level of empathy might affect the pharmacist-patient relationship and result in losing recurring customers over time, which in turn ends in job frustration because of a dissatisfactory income. Hence, the observed loop-cycle of older age leading to continuous job dissatisfaction, which leads to lower empathy.

Our study showed that having a practice experience between 3 years and less than 6 years was significantly associated with lower empathy. Previous studies [9] suggest that professional experience years play a role in the empathetic development process of community pharmacists: those more experienced develop empathetic skills socially, through a social learning model. Thus, pharmacists look up to fellow pharmacists or professional mentors during their formative professional years and use these observed attitudes and behaviors later on in their professional interactions [9]. Therefore, pharmacists having 3–6 years of experience are in transition between learning from experienced mentors and developing their empathetic social skills. These pharmacists are in a middle zone, where they don’t need mentors anymore but lack enough experience to navigate empathetic interactions on their own. Being in this intermediate level can explain why they exhibit low empathy levels when compared to the other groups, keeping in mind that empathy is a social skill, mostly acquired through social practice and interactions with as many patients as possible through time. One way to improve empathy, in this case, is to develop continuous and integrated strategies to enhance empathic skills, and to progressively evaluate the ability of pharmacists to respond empathically throughout their clinical progression [38].

Statistically, our results found no significant relationship between depression score and empathy level. This may seem counterintuitive, but several well-cited studies explain the lack of association between different measures of empathy and depression. O′ Connor et al. [39] suggest that the ability to know what people are thinking, termed Theory of Mind (ToM), is a prerequisite for empathy and is common in depressed people. However, healthy empathy also requires an understanding of causality, which is affectively distorted in depression. Therefore, the presence of depression is not associated with empathy levels since depressed people may have normal cognitive empathy levels, but a different empathy experience (due to the misinterpretation of empathy).

### Clinical implications

This study showed that lower levels of empathy were significantly associated with older age, increased physical and mental work fatigue, and practice experience between 3 years and less than 6 years. No future measures or adopted solutions can affect the age-related factor. However, it is possible to improve work fatigue conditions. One way to decrease fatigue is for pharmacists: to get proper rest, attend to the body’s physiological needs by maintaining balanced lifestyles, ensure good physical health, and receive the support needed for proper mental health. Since empathy is an active dynamic process, the community pharmacist becomes more receptive to patients’ needs after decreasing work fatigue. This way, the pharmacist is better equipped to reach out to patients and fulfill the accepted model of pharmacy practice, which requires effective communication and the use of interpersonal skills.

Pharmacy schools took many initiatives to include curriculum modifications, aiming at enhancing empathy in pharmacists during their training. Indeed, by going to the source as early as possible, pharmacy students get a solid formation on empathy and its psychosocial attributes [40]. Implementing different learning techniques i.e., simulated learning with ethical scenarios, inter-professional and problem-based learning, can lead to enhanced values, ethics, and decision-making [41]. Several studies show that empathetic relationships with patients are followed by rewards, with empathetic dialogue leading to patient satisfaction, adherence to treatment (sometimes), comprehension, and enhanced clinical outcomes [42].

### Limitations

Our study has several limitations. First, information bias may occur since the questionnaire used is based on self-report of pharmacists’ thoughts and interpretation of symptoms. Social desirability bias may also be noted since participants may want to seem conforming to social norms; this was taken into consideration by making sure that they know that their answers remain anonymous. Secondly, no causality inferences can be made since this study is cross-sectional. Further longitudinal studies are warranted to make causality inferences. However, for this study, we were able to make associations (evaluation of factors associated with empathy). Thirdly, some of the scales used in the questionnaire, such as the Toronto Empathy Questionnaire, are not validated in Lebanon. Nevertheless, the results derived from these scales were found to be noteworthy by the authors, since they provide consistency upon comparison to other studies.

## Conclusion

This study shed the light on some factors associated with empathy among Lebanese community pharmacists. Low empathy levels were significantly associated with factors such as age, practice experience, and mental and physical work fatigue, all of which impact the practice, as the accepted model of pharmacy practice requires that pharmacists establish effective communication and use interpersonal skills. Therefore, developing empathetic communication skills is considered essential. Furthermore, increased mental and physical work fatigue should not hinder community pharmacists’ access to self-care, whether for their mental or physical health.
